# Supplementary material for: Enlisting the Ixodes scapularis Embryonic ISE6 Cell Line to Investigate the Neuronal Basis of Tick—Pathogen Interactions
Source: Pathogens. 2021 Jan 14;10(1):70. doi: 10.3390/pathogens10010070 (PMC7828734; doi:10.3390/pathogens10010070)
Supplement: Supplementary file 1 [file pathogens-10-00070-s001.pdf]

Supplementary information

Enlisting the *Ixodes scapularis* Embryonic ISE6 Cell Line to Investigate the Neuronal Basis of Tick-Pathogen Interactions

Lourdes Mateos-Hernández[1], Natália Pipová[2], Eleonore Allain[1], Céline Henry[3], Clotilde Rouxel[1], Anne-Claire Lagrée[1], Nadia Haddad[1], Henri-Jean Boulouis[1], James J. Valdés[4,5], Pilar Alberdi[6,7], José de la Fuente[6,8], Alejandro Cabezas-Cruz[1*], Ladislav Šimo[1*]

[1] UMR BIPAR, Laboratoire de Santé Animale, ANSES, INRAE, Ecole Nationale Vétérinaire d'Alfort, Paris-Est Sup, Maisons-Alfort, France; lourdes.mateos@vet-alfort.fr (L.M-H.) ; clotilde.rouxel@vet-alfort.fr (C.R.) ; anne-claire.lagree@vet-alfort.fr (A-C. L.) ; nadia.haddad@vet-alfort.fr (N.H.); henri-jean.boulouis@vet-alfort.f (H-J.B)

[2] Faculty of Science, Pavol Jozef Šafarik University in Košice, 04180, Slovak Republic; kokosova.natalia@gmail.com

[3] Université Paris-Saclay, AgroParisTech, Micalis Institute, PAPPSO, INRAE, 78350, Jouy-en-Josas, France celine.henry@inra.fr

[4] Institute of Parasitology, Biology Centre of the Czech Academy of Sciences, Branisovska 31, CZ- 37005 Ceske Budejovice, Czech Republic valdjj@gmail.com

[5] Department of Virology, Veterinary Research Institute, Hudcova 70, 62100 Brno, Czech Republic.

[6] SaBio Instituto de Investigación en Recursos Cinegéticos IREC-CSIC-UCLM-JCCM, Ronda de Toledo s/n, 13005 Ciudad Real, Spain maria.alberdi@uclm.es (PA); josedejesus.fuente@uclm.es (JF)

[7] Neuroplasticity and Neurodegeneration Group, Ciudad Real Medical School, Regional Centre for Biomedical Research (CRIB), University of Castilla-La Mancha, 13071, Ciudad Real, Spain

[8] Department of Veterinary Pathobiology, Center for Veterinary Health Sciences, Oklahoma State University, Stillwater, OK 74078, USA jose_delafuente@yahoo.com

* Correspondence: ladislav.simo@vet-alfort.fr; Tel.: +33 1 49 77 46 52 (L. Š.), cabezas-cruz@vet-alfort.fr , Tel.: 0033631235191 (A.C.C.)

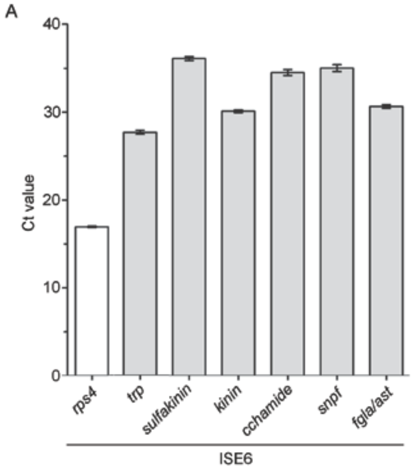

Supplementary figure S1. (A) Relative expression of ribosomal protein S4 (*rps4*) transcript and six different neuropeptide transcripts in uninfected ISE6 cells. The Ct values (i.e., the mean-standard error) for the two biological replicates are shown.

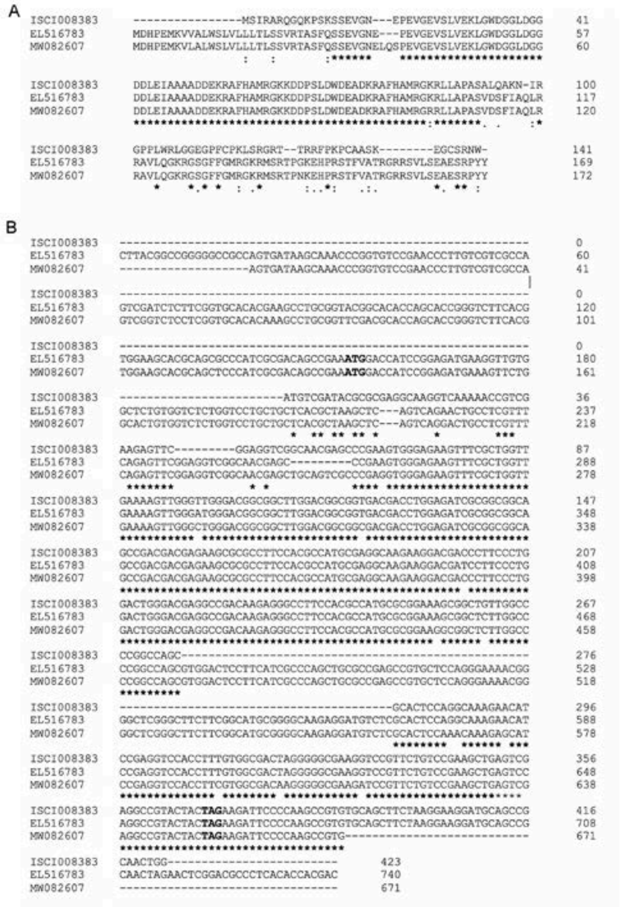

Supplementary figure S2. Protein (A) and nucleotide (B) alignment of *Ixodes scapularis* and *Ixodes ricinus* TRP. ISCI008383 – *I. scapularis* genome-predicted transcript; EL516783 - *I. scapularis* EST sequence; MW082607 – *I. ricinus* transcript (identified in this study). Please note the incorrect N-terminal sequence of ISCI008383 (A) caused by the incomplete 5'-end (B). In addition the shift in the 3'-end (B) reading frame caused an improper conceptual TRP translation (A) of ISCI008383.

Name	Forward primer (5'-3')	Reverse primer (5'-3')
fgla/ast	CACCATGCTGCTGAGCAA	CTACTTTCTCCGGAAGCAGG
cchamide	GTGTCCTCTGACGTGATGC	GTCTATCAAGCCTCGCCTG
short neuropeptide f	GCCTGCATCCTTCTCGATAG	CTTCCGTTCCATGGTGTGG
sulfakinin	CTTTGTCTTCTTGCAGCACTC	GACGGGATCGATCATGTCC
trp	CTTGCGGATGGCATTGGTTC	CACCTATTGGCGTAACCCG
kinin	GAGGCAGGACAAAGAGAGC	CGAGAAGGTGCTGTCCTTAG
natalisin (1st set)	AACACTCGGATCACAGACCC	CATCGTGACGTCGAAAGAGT
natalisin (2nd set)	AGGTAGTATGTCCGACCAAC	ACAGGTGACTACTGCGACAA
natalisin (3rd set)	CTTGCGGATGGCATTGGTTC	CACCTATTGGCGTAACCCG

Supplementary table S1. List of primers used in our qRT-PCR experiments.
